# Treatment of sexual trauma dissolves contamination fear: case report

**DOI:** 10.3402/ejpt.v4i0.19157

**Published:** 2013-01-07

**Authors:** Mirjam J. Nijdam, Marthe M. van der Pol, Ron E. Dekens, Miranda Olff, Damiaan Denys

**Affiliations:** 1 Department of Psychiatry, Academic Medical Center (AMC), University of Amsterdam, Amsterdam, The Netherlands; 2 Arq Psychotrauma Expert Group, Diemen, The Netherlands; 3 Institute for Neuroscience, Royal Netherlands Academy of Arts and Sciences, Amsterdam, The Netherlands

**Keywords:** Obsessive–compulsive disorder, posttraumatic stress disorder, comorbidity, combined treatment, psychotherapy, Eye Movement Desensitization and Reprocessing, pharmacological treatment

## Abstract

**Background:**

In patients with co-morbid obsessive–compulsive disorder (OCD) and posttraumatic stress disorder (PTSD), repetitive behavior patterns, rituals, and compulsions may ward off anxiety and often function as a coping strategy to control reminders of traumatic events. Therefore, addressing the traumatic event may be crucial for successful treatment of these symptoms.

**Objective:**

In this case report, we describe a patient with comorbid OCD and PTSD who underwent pharmacotherapy and psychotherapy.

**Methods:**

Case Report. A 49-year-old Dutch man was treated for severe PTSD and moderately severe OCD resulting from anal rape in his youth by an unknown adult man.

**Results:**

The patient was treated with paroxetine (60 mg), followed by nine psychotherapy sessions in which eye movement desensitization and reprocessing (EMDR) and exposure and response prevention (ERP) techniques were applied. During psychotherapy, remission of the PTSD symptoms preceded remission of the OCD symptoms.

**Conclusions:**

This study supports the idea of a functional connection between PTSD and OCD. Successfully processing the trauma results in diminished anxiety associated with trauma reminders and subsequently decreases the need for obsessive–compulsive symptoms.

The psychopathology of obsessive–compulsive disorder (OCD) and posttraumatic stress disorder (PTSD) shares the need for control as a core theme. OCD patients are obsessed with absolute certainty and try to control their anxiety provoking thoughts with repeated acts or rituals. PTSD patients try to regain control after experiencing an uncontrollable event by avoiding triggers of the event (Olff, Langeland, & Gersons, 2005). OCD and PTSD co-occur when a patient tries to control recollections of a traumatic event by means of obsessive–compulsive symptoms. It has been presumed that obsessive–compulsive symptoms may function as a coping strategy (Gershuny, Baer, Jenike, Minichiello, & Wilhelm, 2002), conveying the illusion of control in the midst of an uncontrollable event and its aftermath (Pitman, 1993).

Research into traumatized clinical samples has confirmed that OCD as well as PTSD may emerge after exposure to traumatic events, and that the content of the obsessive–compulsive symptoms may be associated with the trauma (De Silva & Marks, 1999; Gershuny, Baer, Wilson, Radomsky, & Jenike, 2003; Pitman, 1993; Sasson et al., 2005). Whereas the lifetime prevalence of PTSD is 7.4% (De Vries & Olff, 2009), the prevalence of PTSD in treatment-seeking OCD patients varies between 10.2% (Shavitt et al., 2009) and 39.4% (Gershuny et al., 2008), and the prevalence of OCD was found to be 40.9% in a sample of terror-and combat-related PTSD patients (Nacasch, Fostick, & Zohar, 2011). A case series in treatment-resistant OCD patients suggests that the co-occurrence of OCD and PTSD may hamper the effectiveness of treatment (Gershuny et al., 2003). In this case series, a treatment-induced decrease of OCD symptoms evoked an increase of PTSD symptoms, and vice versa an increase of OCD symptoms led to a decrease in PTSD-specific symptoms.

## Objective

To this point, the sparse literature on concurrent OCD and PTSD has primarily been focused on determining comorbidity rates and on the impact of a trauma or PTSD on treatment response in OCD. In clinical practice, however, it is often very difficult to know what to treat first, OCD or PTSD? The aim of the current study is to examine whether treating PTSD by targeting the traumatic event facilitates OCD treatment. We describe an outpatient with OCD and PTSD who underwent trauma-focused psychotherapy in addition to exposure and response prevention (ERP) treatment and pharmacotherapy.

## Case report

Albert, a 49-year-old Dutch man who lives with his male partner, was referred to our department by his company doctor after 6 months of sickness absence from his work as a teacher. At intake, he met DSM-IV criteria for PTSD and OCD with daily trauma recollections, obsessions, and compulsions of cleaning and ordering for many hours a day. The trauma took place at the age of 14 when a 32-year-old man raped Albert. The PTSD symptoms started at the age of 21, when he first discussed what had happened with his partner. His OCD symptoms started at the age of 34, when he told a therapist about the trauma. Trauma recollections then evoked thoughts in his mind about feeling “dirty” (including his own body and surroundings). He tried to get rid of them by repeatedly showering, cleaning, exercising, and meticulously arranging things in his house.

After intake, paroxetine was started and increased to 60 mg, which improved his sleep and made him feel less anxious, though the diagnoses of PTSD and OCD remained. After 3 months, Albert received Eye Movement Desensitization and Reprocessing (EMDR) therapy in a randomized clinical trial (Nijdam, Gersons, Reitsma, de Jongh, & Olff, 2012). Written informed consent was obtained after the nature of the procedures was explained. The study design of the trial was approved by the Institutional Medical Ethics Committee and was performed in accordance with the Code of Ethics of the World Medical Association (Declaration of Helsinki). EMDR is a psychotherapeutic method in which the most distressing images of the traumatic event are identified and processed consecutively (De Jongh & Ten Broeke, 2004). Albert received nine psychotherapy sessions, of which seven consisted of EMDR. From session 6, when the PTSD symptoms markedly decreased, ERP techniques were added to tackle the remaining OCD symptoms.

Albert's symptom course was closely monitored by means of widely used, reliable and valid instruments, as displayed in Table 1. Albert reported that the rationale for his obsessive–compulsive behavior ceased as soon as the trauma impact disappeared. The images of the traumatic event dissolved and he realized that he was not “dirty”, he felt less anxious, and was less compelled to perform his rituals. After 1 year, during which paroxetine treatment was continued, Albert is still free of PTSD and OCD symptoms (Impact of Event Scale—Revised total score 3, Yale-Brown obsessive compulsive scale total score 0).

**Table 1:** Albert's clinical diagnoses and symptom course before and after EMDR + ERP treatment

Measure	Baseline (*t*=0)	After EMDR + ERP (*t*=9 weeks)	Follow-up (*t*=17 weeks)
PTSD diagnosis^a^	Yes	No	No
OCD diagnosis^b^	Yes	No	No
PTSD symptom severity^a^ (clinician-rated)	33 (severe PTSD)	17 (moderate PTSD symptoms)	7 (subclinical PTSD symptoms)
PTSD symptom severity^c^ (self-reported)	79 (severe PTSD)	2 (subclinical PTSD symptoms)	0 (no PTSD symptoms)
OCD symptom severity (clinician-rated)^d^	18 (moderately severe OCD)	Not available	1 (subclinical OCD symptoms)
Obsessions	9		0
Compulsions	9		1
Depressive symptom severity (self-reported)^e^	4 (mild depressive symptoms)	0 (no symptoms)	0 (no symptoms)
Severity of general anxiety symptoms (self-reported)^e^	7 (moderate general anxiety symptoms)	0 (no symptoms)	0 (no symptoms)

a Structured Interview for PTSD, total possible score ranges from 0 to 68 (Davidson, Malik, & Travers, 1997)

b Structured Clinical Interview for DSM-IV disorders (Spitzer, Gibbon, Janet, & Janet, 1996)

c Impact of Event Scale – Revised, total possible score ranges from 0 to 110 (Weiss & Marmar, 1997)

d Yale-Brown Obsessive Compulsive Scale, total possible score ranges from 0 to 40 (Goodman et al., 1989)

e Hospital Anxiety and Depression Scale, total possible score ranges from 0 to 21 (Zigmond & Snaith, 1983).

## Discussion

This case report demonstrates a successful treatment of comorbid OCD and PTSD, which hitherto has not been described in literature. We report on an outpatient with moderately severe OCD and severe PTSD, whose obsessive–compulsive symptoms commenced after revealing a sexual assault and were maintained by recollections of the trauma. The OCD and PTSD symptoms disappeared completely following a combined treatment with paroxetine and nine sessions of EMDR and ERP.

Our treatment strategy was based on the hypothesis that OCD symptoms serve as a strategy to cope with reminders of the trauma, which has been put forward by previous studies (Gershuny et al., 2002; Pitman, 1993). Unlike previous studies, PTSD symptoms in our study were targeted first with trauma-focused psychotherapy, followed by treatment of the OCD symptoms with ERP techniques. Applying EMDR before ERP made it easier for the patient to reduce OCD symptoms because of decreased anxiety to trauma reminders. Our findings are in line with another case report that successfully applied trauma-focused cognitive behavioral therapy in combination with ERP techniques in PTSD with comorbid OCD symptoms (Tuerk, Grubaugh, Hamner, & Foa, 2009). The addition of EMDR, however, to ERP and SSRI has not yet been described previously. This case raises the question whether EMDR can be beneficial for OCD patients without a PTSD diagnosis. EMDR is increasingly applied for psychiatric disorders other than PTSD, if one or more causational events can be identified in their etiology. Mental images are reported by 81% of OCD patients, which often consist of memories of earlier adverse events (Speckens, Hackmann, Ehlers, & Cuthbert, 2007). The onset of OCD has been linked to an increased number of life events (McKeon, Roa, & Mann, 1984) and stressful events characterized by significant loss or increased responsibility (Rasmussen & Tsuang, 1986). With EMDR, mental images of those events could be targeted and their emotional distress can disappear.

## Conclusions

To sum up, the current study supports the idea of a functional relationship between PTSD and OCD and suggests that trauma-focused psychotherapy may be useful in cases of concurrent PTSD and OCD originating from a traumatic event. By successfully processing the trauma, anxiety to trauma reminders decreases which may facilitate ERP treatment for OCD. This can lead to a shortened treatment trajectory and possibly an improved long-term treatment outcome. The findings are too preliminary to support widespread dissemination. Therefore, further research is encouraged to confirm these findings.
